# Popular Illustrations of Medicine

**Published:** 1830-04-01

**Authors:** 


### Medico-Chirurgical Review,

### No. XXIV.

JANUARY 1, TO APRIL 1, 1830.

### XV.

**Popular Illustrations of Medicine.** By *Shirley Palmer*, M. D. 8vo. pp. 396. Baldwin, 1829.

Falsa vincere veris.

We shall not here discuss the long agitated question, whether mankind is or is not benefited by the dissemination of *popular* works on medicine. No physician of observation can fail to perceive that the practice of physic is thereby increased. In the history of each case, it generally comes out, incidentally of course, that the patient has been tampering with his complaint for some days before the doctor is called in; trying such and such a remedy prescribed in such or such a work on popular medicine. By this procedure, much precious time is lost, and many days or weeks added to the duration of the malady. But this is not the question. Can an author who professedly writes for *popular* instruction, expect that his work shall be reviewed or noticed in strictly professional Journals? We think he ought not to expect it—and yet he invariably does so. He addresses his lucubrations *expressly* to the *general* reader—and expects that the *medical* Reviewer should carefully analyze them and delineate their contents to the profession! Really this is a very unreasonable expectation; and it is one which we have never hitherto gratified. The appearance, however, of a *popular* treatise on medicine from the pen of our old friend and collaborateur, Dr. Palmer, startled us not a little—for we thought he was one of the last men on earth who would have embarked in such an undertaking—an undertaking almost invariably originating in knavery—the lowest species of literary speculation—or disguised quackery. And, indeed, it is only when based on one or other of these principles, that Popular Medicine is ever successful. We were quite satisfied that Dr. Palmer would never condescend to quackery or puffery—and from the moment we read the title-page we had misgivings that *he* was not the man for a popular treatise on diseases. A glance over the work has satisfied us that it is a complete failure. It is infinitely too learned, too scientific, and too technical, for the general reader; while his title-page and dedication tend to prejudice the medical profession against the work. Who would expect a popular treatise on medicine from the pen that gave vent to the following passage?

"Every attempt to instruct the public in the discrimination and management of particular diseases, must be as futile in execution as absurd in principle. No one professes to rectify the derangements of even an ordinary piece of mechanism unless he be duly qualified by previous education, for the undertaking.

Who then, unprovided with the light and the instruments of science, shall presume to repair the injuries, or regulate the deviations of a machine, the structure of which is far more delicate and complex than that of any work of human production."—*Preface.*

A little further on Dr. Palmer endeavours to excuse or rather to justify the "attempt to instruct the public," by saying that "civilized man should be taught, by a clear exposition of their source, character, and operation, to protect himself from the influence of the various EXCITING CAUSES OF DISEASE, and thus avert the sufferings which they may otherwise inflict." Certainly, if the causes of diseases were always or even generally cognizable by the senses, and could be portrayed to the non-professional world at large, it would be very meritorious in any physician to do so. But who does not know that ETIOLOGY is one of the most difficult and mysterious branches of our *occult* science? Independently of this, Dr. Palmer does not confine himself to the *exciting causes* of diseases, but goes far more deeply into their pathology, treatment, &c. Then comes another hacknied excuse for POPULAR MEDICINE—the attempt to set up the patient as the judge of his doctor's medical knowledge!

"He should, at least, know so much as may enable him to elicit from his professional attendant, and to comprehend, an explanation of the nature of any disease under which he may suffer; and the leading principles of the treatment to which he may be required to submit. And surely the talent or integrity of that practitioner may be fairly questioned, who cannot render his views and opinions intelligible to a common intellect." vi.

Verily, Dr. Palmer, you would have some difficulty in rendering your "views and opinions" intelligible even to your own brethren—and much more to "common intellects," who, we venture to assert, will not comprehend one line in every five hundred throughout the volume! In short, Dr. Palmer is totally unqualified for the task of a "*popular*" writer on medicine, from entire want of that trickery, quackery, mendacity, ignorant presumption, and utter disregard of every thing but pelf, which characterise the writings of nine-tenths of this class of authors. A cursory look over the volume before us has, however, convinced us that it is far better fitted for professional than popular perusal—at all events, we hope to prove, in the course of this article, that there are many facts, views, observations, and even opinions, in Dr. Palmer's *Popular* Treatise, which are deserving of notice in a strictly *medical* Review. Our author, indeed, informs those of his professional readers "who shall condescend to peruse these pages," that they may, perchance, descry some bold deviations from commonly received opinion respecting the causes and the treatment of several diseases. Dr. Palmer was, we believe, a strenuous disciple of the Abernethy school, and flourished the CHYLOPOIETIC BESOM very freely in sweeping diseases out of the midland counties. But "ten years of close and extended observation have elapsed since a great revolution was effected in the writer's early views. Subsequent experience, while modifying and correcting, has served only to confirm the change insensibly wrought upon his mind by the irresistible evidence of facts." We shall introduce, further on, a severe, but not unjust, critique on the EXTRAVAGANZAS of the Bartholomew school, which are now beginning to be seen in their proper light, but not before they have inflicted their quota of misery and perhaps death on the human race!

The work is divided into ten chapters, from each of which we hope to glean something that may afford food for useful reflection, if not direct and positive information.

The first short chapter glances at the various Exciting Causes of Disease. In this chapter, which is little more than introductory, Dr. P. shews that his attention has been strongly directed to the operation of *moral* causes in the production of corporeal maladies—and he strongly regrets that writers on popular medicine should have neglected this important branch of etiology. But, if Dr. P. had thought for a moment, he would have recollected that these popular writers knew nothing of the matter!

In the second chapter, Dr. P. considers the pre-eminent importance of the brain and spinal marrow in the animal economy—and shews himself to be a temperate and tolerant examiner of the doctrines of phrenology.

"Specious beyond all precedent, and apparently confirmed and illustrated by many striking facts and occurrences in the world around, Phrenology assumes a most imposing character, and presents a powerful claim to public attention. Yet the painful remembrance of many a past illusion, once equally bright with promise to human fortunes and improvement, inculcates a salutary lesson of circumspection. On the one hand, too strange and splendid, and, in the language of a gifted writer,* too 'good,' to be true in all the minuteness of its details, the theory of the German philosophers will yet be regarded by the reflecting mind as, on the other, too ingenious,—too clearly illustrative of several otherwise inexplicable phenomena exhibited by man in his various passions, propensities, and diseases,† to be utterly destitute of foundation in nature. The introduction of error into their practical application can never be fairly or logically adduced as a proof of unsoundness in the principles of a science, especially where that science, yet in its infancy, is surrounded by extraordinary sources of doubt and difficulties of investigation. Whatever be the ultimate fate of their doctrines, much is due to these celebrated men for the valuable additions which they have unquestionably made to the previously imperfect knowledge of the human brain; and for the unbending spirit with which they have submitted to toil and indignity in the promulgation of their discoveries and opinions. The generous mind sickens with pain and humiliation on reflecting that, in a civilized land, and in a city boasting of her pre-eminent elevation in literature and philosophy, a zealous and enlightened stranger should have encountered, for a while, nothing but obloquy and ridicule. Respect, at least, is justly claimed by all those, however visionary their doctrines, who, inspired by a love of science, have unsparingly sacrificed to its promotion, their home, their fortune, and repose. And History, in retracing the progress of the human mind during the nineteenth century, will indignantly avenge the insults, with which an invidious contemporary has attempted to slur the distinguished names of Gall and Spurzheim; and do ample justice to the splendid talents and attainments, which he, in an evil hour, has affected to despise." 14.

---

* "Dr. Archibald Robertson."

† "The occurrence of partial insanity,—of mental hallucination on one exclusive subject,—appears to be quite inexplicable on the supposition that the whole of the brain constitutes a single organ. And many striking facts in nature and in the phenomena of injury and disease, may be adduced to demonstrate the close connection which exists between the cerebellum and the passion of physical love. Phrenology, moreover, supplies foundations for a system of moral philosophy, more solid and intelligible than any which has hitherto been proposed."

The foregoing and the first specimen of Dr. Palmer's style, will convince our readers that HE is not to be classed among the common herd of writers, or rather ignorant *compilers* of POPULAR MEDICINE.

After some cursory observations on the structure and functions of the brain and spinal marrow, Dr. P. remarks that, as the substance and membranes of the brain may be the seat of congestion, inflammation, and other diseases, without increased sensibility, enlargement, or other alteration of the cranial bones—so different diseases of the spinal marrow may exist, and even proceed to a fatal termination, without any unnatural tenderness, projection, or other appreciable change of the bony canal. Men of great reputation and experience have been known to deny the existence of spinal disease, because, on examination, they could not detect any morbid condition of the bones.

Inflammation of the brain and spinal marrow, when acute, is commonly signalized by strongly marked phenomena—not so some chronic affections of both organs, which proceed in such an insidious manner, or assume such Proteiform characters, as to elude the scrutiny of the most vigilant physician, if his attention have not been previously and powerfully excited to this obscure tribe of cerebro-spinal diseases. Dr. P. elucidates these observations by allusion to one or two affections which, although very prevalent, are too frequently misunderstood and consequently mistreated.

"In females at an early age, and especially in those of feeble and delicate constitution, pain about the convexity of the sixth or seventh rib, frequently forms a most obstinate and distressing symptom. It is usually, yet not invariably, seated in the left side. In general, it is dull, wearying, confined to a small spot, and distinctly circumscribed. But it is subject to paroxysms of such acuteness and severity, as to assume an aspect very strongly resembling that of *Neuralgia* or tic doloureux; and, without doubt, as closely allied to it in origin as in character. During the violence of these paroxysms, the pain sometimes strikes upwards in the direction of the breast-bone and shoulder; and a sense of numbness, of tingling, and loss of power, are felt in the corresponding arm. Long preservation of the erect posture, fatigue, grief, anxiety, and all the depressing agents, and ingestion of food, particularly in a solid state, are constantly followed by aggravation of the pain. It suffers no very decided change on full inflation of the lungs with air; nor is cough or difficulty of breathing, although sometimes forming a dangerous complication, essentially connected with it. By the assumption of the recumbent posture, great relief is almost instantly obtained: and, so signal and invariable are the results of this simple expedient, that it appears to constitute the best, if not the only, diagnostic sign of the peculiar character of the affection.

"Females, whose time is unduly devoted to fatiguing domestic occupations or sedantary pursuits, and who are consequently excluded from the invigorating influence of air and exercise, most frequently suffer from this "intercostal pain." Head-ache, depression of spirits, debility and lassitude of the muscular system, weakness of the pulse, tremor or palpitation of the heart, wearying pains and coldness of the extremities,—constitute its ordinary,—and excessive torpor of the bowels, one of its most invariable—attendants.

"It is occasionally complicated with loss of voice or *Aphonia;* and sometimes, although more rarely, with unnatural pulsation in the chest and obstinate rejection, by vomiting, of every alimentary substance introduced into the stomach. Instances of its obvious alternation with intense pain in the head, accompanied by violent throbbing of the carotid arteries, and by all the phenomena of a high state of cerebral congestion, have sometimes been observed.

"Commonly regarded as organic disease of the heart, or inflammation of the pleura, liver, or spleen, the treatment of this affection is, in most instances, as unsuccessful, as the views upon which it is founded, are erroneous. Blood-letting, general and local, blistering of the side, mercury, abstinence, and all the depleting remedies, afford sometimes a transient, but delusive, mitigation. More frequently, they aggravate the pain, and increase the debility by which it is constantly attended. If blindly persevered in, they may produce irrecoverable exhaustion of the system, or affections more perilous than that which they were intended to relieve. On the other hand, the pain will be tranquillized, and, in general, ultimately subdued by undeviating perseverance in a plan of treatment, of which reclination on a couch for several hours daily, simple but generous diet, aloetic aperients, preparations of iron, and the other metallic and vegetable tonics, stimulation of the integuments of the spine, cold sponging or the shower bath, form the principal constituents." 24.

In the earlier periods of the complaint, Dr. P. observes, the spine, on examination, will evince little alteration from its healthy state. In general, however, a slight degree of tenderness will be evinced on the application of pressure, in one or more points between the middle of the dorsal portion and the summit of the column. At a more advanced period, spinal pressure will scarcely be borne, and is often productive of pain, darting like an electric shock into the chest or arm. According to the duration or severity of the complaint, one or more of the vertebræ will exhibit the well-known characters of disease, and the process of spinal deviation will have commenced. The progress of the morbid changes will be greatly accelerated by debilitating treatment. "Irretrievable distortion of the spine, with all its hopeless and distressing infirmities—habitual hysteria or chorea, nervous atrophy—or pulmonary consumption, are generally the sad consequences of neglect or error of this most common and insidious affection."

Aphonia is a morbid phenomenon of frequent occurrence in delicate or weakly females—and is, according to our author's views, "obviously connected with spinal irritation and congestion, and dependent, for its immediate production, on a morbid state of the inferior laryngeal or recurrent nerves." When of recent origin, Dr. P. has always found it yield to the invigorating plan of treatment. A blister applied to the back of the neck signally expedites the recovery. Mercury he thinks inadmissible, for although it sometimes removes the aphonia, it weakens the patient and renders her more liable to relapse.

We now come to a series of remarks which are evidently issued forth by our talented author with the professed view and confident expectation of contributing effectually towards a counter-revolution in the medical world of these islands, by giving a death-blow to the "DIGESTIVE ORGAN" doctrine and practice, so long supported by the names and authorities of Abernethy, Hamilton, Curry, and a host of their disciples. The author must be allowed to speak pretty freely for himself on this occasion.

"The other disease, which may be most aptly selected as illustrative of insidious cerebral irritation, has, of late, acquired a great ascendancy in these islands. Keeping pace with the rapid progress of intellect and refinement, of commercial speculation and adventure, it almost rivals in frequency tubercular consumption itself; and constitutes, in fact, one of the prevailing chronic diseases of the age. This affection consists in an obscure state of irritation of the brain, commonly implicating the superior portion of the spinal marrow. The causes, which predispose the system to its attacks, are probably connected with some original peculiarity in the moral or physical constitution of the individual. Those which excite it into action, may be distributed into the internal and external. They operate either directly by inducing an increased afflux of blood to the brain, as inordinate mental exertion, anxiety, grief, and the infliction of mechanical violence ; or indirectly by the stimulation of a remote organ, as intemperance ; or by destroying the balance of the blood's circulation, as profuse hæmorrhage. In the majority of cases, however, the disease may be distinctly traced to a moral source. For its proximate cause it apparently depends on augmented impulse, or irregular distribution of blood to the whole, or part, of the cerebral mass. Whatever these causes be, when once induced, it will be kept up, and aggravated, by every moral or physical irritation or excess.

"In its commencement and early progress, this affection is often so obscurely marked as to be with difficulty recognizable. The external phenomena to which it gives rise, are, indeed, sometimes more clearly discernible in a remote organ, than in the brain itself: so that, until it has acquired, from duration, a certain intensity, and until all the ordinary methods of treatment have been found unavailing, the precise seat and character of the disease are rarely suspected. Little known in the tent of savage, or in the peaceful dwelling of pastoral life, it visits the retirement of literature and science, the busy haunts of commerce and refinement. The sedentary in occupation, the ardent and susceptible in feeling, and the highly gifted in intellect or attainment, are peculiarly, although not exclusively, exposed to its aggressions.

"Two distinct varieties of this formidable affection may be clearly discerned in practice. Anxiety of countenance, an air of peculiar restlessness and despondency, obscure pain or heaviness, with increased heat of the head, undue pulsation of the carotid arteries, and slight puffiness of the cheek,* constitute the leading external signs of the complaint in its more simple and concentrated form. The tongue is usually clean; the secretions of the liver natural; the bowels torpid; the appetite unimpaired ; the pulse at the wrist feeble, and somewhat accelerated. The mind is haunted, especially at night, by gloomy anticipations and

---

* "There are a few external signs which, although very commonly present and distinctly marked, have not been generally observed, or recognized, as diagnostic of derangement of function, or disease of structure, in the brain. One of these is *a slight œdema, or pitting of the cheek on pressure.* It very often exists in simple congestion; and, in such cases, has repeatedly been pointed out by the writer to his professional friends. The mode of its production is not very obvious. It must not be confounded with the swelling of the face, very frequently observed in diseases of the heart. The general character of the attendant symptoms, and the absence of dropsical swelling of the extremities, will suffice to prevent the circumspect practitioner from confounding it with the latter more decided and ominous appearance. *Obscure pain with stiffness of the muscles of the neck,* constitutes another of these neglected signs. It usually accompanies those cases, where mental restlessness and excitement, dependent on cerebral irritation, occur at intervals ; and the access of the paroxysm is then marked by recurrence or aggravation of it. Wrinkling of the integuments of the forehead, produced by contraction of the frontal muscle, is its general attendant. By hasty or unreflecting observers, it is often beheld, and treated, as a rheumatic affection. The last diagnostic sign, to which it will here be necesary to call the reader's attention, is an *unnatural and most striking projection of the eye-ball.* The progress of this change is, in most instances, slow and uninterrupted. In others, its accession has been more sudden. Its extent is sometimes such that the organ seems as though it were partly protruded from the socket. The practical inferences, which may be drawn from a decided existence of this sign, are as unerring as unfavourable. It will be invariably found to indicate that a fatal process of morbid alteration is going on within the skull."

terrific images. In some severe cases, there occur intervals of respite from suffering, the duration of which is as uncertain as their cause inexplicable; and the recurrence of the paroxysm is generally preceded by involuntary starting of the muscles of the spine; pain, and stiffness, of those of the neck; and attended with extreme restlessness, and flushing heat of the head and face. Its ordinary termination, when mistaken or unsuccessfully treated, is organic disease of the brain, and insanity, paralysis, or fatal apoplexy.

"In the other yet more obscure and embarrassing shape, which the complaint frequently assumes, one or more of the remote organs conspicuously exhibit the signs of its existence. It varies in aspect according to the organ thus consecutively affected at the period of observation: and the diagnosis and treatment of the disease are too often erroneously fixed by an exclusive view to this imagined source. By writers apparently unacquainted with its actual origin, it has been aptly described as in form a real Proteus; in character, a genuine Mimosis. The lungs, the heart, or uterus, frequently suffer in this insidious affection: and the leading symptoms will be obviously determined by the nature and function of the organ thus implicated. Hence, dry irritating cough and disordered breathing; palpitation of the heart and faintness; and violent hysteria, constitute the respective signs of the three different secondary irritations just specified. They may exist separately or together;—alternate with hiccup and diverse convulsive affections of the muscular system;—and sometimes succeed each other with such rapidity and violence as to astonish and perplex the practitioner who has not acquired a correct knowledge of their source and treatment. But on different portions of the intestinal canal, particularly the stomach and the colon, this cerebral irritation most frequently exerts its baneful influence: and, here, as in all other cases of complicated derangement, the consecutive re-acts upon, and aggravates, the primary complaint. The disease, thus established, may continue very long without inducing change of structure in the organ sympathetically disordered, or in its original seat, the brain itself. After having embittered, for months, or even years, the existence of the unfortunate patient, and too often rendered him an object of unmerited ridicule or reproach to the thoughtless or unfeeling, the disease is removed either by some auspicious change in his external circumstances, or by an interior revolution, spontaneously or artificially accomplished. Most commonly, however, neglected or mistaken, it pursues a fatal progress. The disordered function of the organ consecutively affected, terminates, at length, in incurable alteration of structure;* or, the signs of this derangement suddenly disappearing, the morbid action becomes concentrated within the brain itself, and exhibits all the external characters of the variety, first described, in an aggravated form. Softening of its substance and extravasation of blood; or inflammation with effusion, or thickening or ossification of the membranes of the organ ensues: and death terminates the conflict; or a state of madness or imbecility, far more terrible than the grave, obscures, or for ever paralyses, the intellect of the wretched sufferer." 32.

This is the far-famed "DISORDER OF THE DIGESTIVE ORGANS," a title which, Dr. Palmer avers, has been almost universally and indiscriminately applied to two very different affections—"different in source, though somewhat analogous in external character." Our author then dedicates a chap-

* "This fact is clearly illustrated by the case of the celebrated exile of St. Helena. He died from an organic disease of the stomach, induced, not as Dr. Kinglake whimsically argues, by the immoderate use of snuff, but by the incessant agitations of a singularly powerful and restless brain. This extraordinary man had never committed dietetic excesses. It will generally be found that persons, destroyed by cancerous affections or tumours of the stomach, have possessed strong passions, or suffered great mental conflict or solicitude."

ter to the origin and progress of the "intestinal school"—to an exposition of the errors resulting from the extravagant application of its doctrines—the "injury which it has inflicted on the cause of science—and the signs of its approaching fall."

Dr. Palmer is as well aware as any of our readers, that, for years past, we have been pointing out the mischiefs which daily occur in practice from the indiscriminate use, or rather abuse, of strong and long-continued purgative medicines. We shall endeavour to make room for an extract or two from the anti-Abernethian chapter, which is written with considerable spirit. After pointing out the failure, and even the dangers, of Dr. Hamilton's system, when carried too far in chorea and hysteria, when, "by abstraction of blood from the head and blistering of the spine, an occasional aperient, ammonia, prepations of iron, and the shower-bath, the disease may, in general, be far more promptly and permanently subdued," Dr. P. turns to the intestinal school of Bartholomew's.

"To the opinions and practice of Mr. Abernethy, however, it is especially important to direct the attention of the professors of medicine and the public. Such are the genius and reputation of this extraordinary man;—such the eloquence and enthusiasm with which he has promulgated his intestinal doctrines;—so specious is the theory which he has constructed,—so easy of adoption the practice which he inculcates;—that, upon foundations, far better calculated for the more transient erection of a fortune and a name, a school has, at length, arisen. The success of these doctrines has been as unprecedented as their simplicity;—their errors as signal as the fate which they are, ere long, destined to experience. To deny that vigilant attention to the state of the intestinal canal really involves an admirable principle in regulating the disorders of the general health, and of the various organs with which that canal so intimately sympathizes, would argue blindness as desperate as that which distinguishes the more bigotted adherent of the intestinal school. Yet that, from inordinate attachment to these fashionable and seductive doctrines, have arisen carelessness in the observation, and impotence in the treatment, of diseases, no candid or experienced observer can hesitate to admit. If all the various derangements of the human fabric result from irritation or disturbance of one organ or system of organs;—if all the morbid phenomena, which signalize these derangements, be referrible to one source, and curable by one plan of treatment slightly modified,—how greatly may the study of medicine be curtailed, and its practice simplified. The time, labour, and fortune, heretofore expended upon medical education, have obviously been expended in vain. The importance of minute investigation into the history, and seat, and character of diseases, and the external signs by which they may be respectively distinguished during life, vanishes, like a morning-dream, before the light of the new doctrines. The illiterate empiric and the village crone may again, as in days of old, enter into successful competition with the enlightened man of art. Obscurity in the nature, and hesitation in the name and treatment, of an individual affection, can no longer exist. The intestinal discharges and the tongue of the patient alone require inspection. To mercury and sarsaparilla, senna and the neutral salts, the catalogue of medicinal remedies may be safely, and most conveniently, restricted. And those stubborn diseases, which now and then inexplicably defy the united operation of blue pill, rhubarb, and the vegetable decoctions, may be confidently denounced as incorrigible; and their unhappy victim be abandoned, without further struggle, to his fate." 41.

Dr. P. is afraid that Nature, in her various operations, however simple and beautiful, does not act with such uniformity as may suit the generalizing spirit, and the sweeping inferences of the Abernethian theory. Man, in the progress of civilization, and in the lapse of ages, has deviated widely from the simple path which Nature originally pointed out:—his diseases have, consequently, assumed the artificial character of his habits, and have become more complex and obscure. No doubt too, that man possesses various other organs besides stomach, bowels, and liver—all as important—and many of them as susceptible of disorder. The disciple of the "Intestinal School," Dr. Palmer thinks, is apt to overlook the brain and spinal marrow—the heart—and the lungs—though each of these may become the seat of primary disease, and propagate it in other organs.

"If, again, it be conceded, as truth demands, that, in these luxurious times so peculiarly favourable to their development, morbid conditions of the stomach do very generally prevail,—the inference, by no means, follows that *all* such affections originate in the organ itself. On the contrary, it must be obvious to the unprejudiced eye, that *many* of these derangements, heretofore considered as primary, are, in fact, merely symptomatic,—consequent on irritation propagated from a distant organ to the stomach; and hence susceptible of permanent removal only by means directed to the source from which they have emanated. If, for instance, the brain, from intense thought, anxiety, or the infliction of external violence, exhibit that state of congestion, which such causes notoriously induce, the digestive organs will immediately sympathize with it, and torpor or derangement of their functions necessarily result. Irritation from a diseased kidney or uterus, will exert such influence on the stomach as to excite all the phenomena and consequences of the most distressing sickness or indigestion. The physician who, under these circumstances, should direct his views and treatment exclusively to the consequent affection, would acquire as little reputation for sagacity, as claim to confidence or success." 43.

We are perfectly willing to accord to the justness of these observations; for who is not aware of the continual action and reaction of one organ on another? That there is a constant tendency in the human mind to generalize too far and too fast, so as to fix the primary seat of diseases in particular organs, there can be no doubt. The difficulty is to distinguish the original from the secondary affection. And it appears to us that the drift of Dr. Palmer's doctrines is to carry the multitude, who do not think too deeply for themselves, into the opposite extreme of the Chylopoietic school—and to locate the great origin of diseases in the brain and spinal marrow—those primary organs of sensation and motion, from whence issues all nervous influence—and on which all functions depend. There would probably be as much danger from running into the extreme of this doctrine (the cerebro-spinal) as that of the one which it is meant to supersede—the gastro-intestinal. The two greatest sources of corporeal disorder are, *moral emotions* of the mind, acting through the channel of the brain and its prolongations—and *improper regimen*, acting through the line of the digestive organs. The most talented practitioners and the most accurate observers will often be puzzled and unable to determine through which of these great channels the *causes* of disease have made their first approach—and how much more will the great mass of routine practitioners, who have hardly time to run from patient to patient, be embarrassed in the discrimination! Very subtle and wire-wove distinctions and discriminations are very beautiful and entertaining on paper—but when they are brought to bear at the bed-side of sickness, even by many of the first-rate manufacturers, we have seen them, like certain other creatures of the imagination—

"Spread their light wings, and in a moment fly;"—

Or, if pressed into the service, and compelled to *work* in actual practice—sad has been the *work* they have put out of hand! It is far better then, we think, to be guided by the more plain and obvious indications—*the symptoms of diseases actually present*—than by fine-spun speculations that are too often as unsubstantial in their origin as they are inefficacious in their application.

Of the two *extremes* then,—and we maintain that the great mass of medical practitioners will be in extremes, whenever a theory is in active operation—we believe that which traces the origin of diseases to the "DIGESTIVE ORGANS" will be less injurious and less harrassing to the public, than that which makes the brain and spinal marrow the seats of the *same maladies.* Let us reflect a moment on the consequences of this last theory, should it ever become fashionable. A practitioner sets out in the morning, and finds three fourths or more of his patients labouring under what have been erroneously called "disorders of the digestive organs," but which recent and accurate observation has traced to irritation or inflammation of the brain and medulla spinalis. Instead of ordering blue pill at night and black draught in the morning, he necessarily and consistently orders them to bed or to a hard sofa, where their heads are to be shaved, their temples leeched, and their spines blistered! On this system, we shall soon have town and country one great hospital! The lawyer may throw up his brief, for who would trust a man to plead a cause, whose brain and spinal marrow are diseased—the merchant may close his counting-house, for the doctor will not permit him to look into his ledger, lest he still farther irritate the brain and spinal marrow—the linendraper, the man-milliner, the confectioner—nay every tenant of the bazar, may shut up shop, for each and every of these labour under the falsely denominated "*disorder of the digestive organs*"*!* According to the old doctrine, the above-mentioned and various other classes of society may still hobble about and look to their affairs, even with a dose of Honest Johnny's black broth in their stomachs, because the said broth has no tendency to *confine* its admirers to the sofa, but rather disposes even the most sedentary habits to brisk loco-motion. Dr. Palmer, indeed, may say that this would be carrying his doctrines to *extremes.* We ask him does he not address a profession which has carried and does carry the other doctrine to extremes—and will always carry a new doctrine, when once embraced, to extremes! All we say is, that the cerebro-spinal doctrine, should it ever become half so epidemic as the chylopoietic, will be productive of ten times more distress to society at large, not only on account of the alarm which must necessarily be created in the minds of the patients and friends by the announcement of cerebral or spinal disease—but on account of the serious inconvenience, not to say ruin, which must result from the system of confinement enjoined among people who have the cares of a family or of complicated commercial concerns hanging over them! Dr. Palmer again may ask, "what has all this to do with a philosophic inquiry after truth?" We answer that, in the first place, we have some doubts about the TRUTH of the matter when found, or supposed to be found. In the second place, it is with physic as with valour—the better part is DISCRETION! How often have we to deplore the lamentable lack of this last, among men of gifted talents and high acquirements in our profession! We every day see more misery inflicted on patients and their families by the indiscreet opinions and directions of their medical attendants, (whose words are oracles in the hour of sickness and danger,) than there is benefit conferred by even the most judicious counsels! We shudder to think on the quantum of mischief that must ensue, when a false and dangerous theory is put into the hands of the multitude in addition to the want of discretion which we here bewail! We have thrown out these hints, not as enemies to free discussion and philosophic inquiry; but with the sincere hope that we may benefit our junior brethren by directing their attention to the cultivation of *wisdom* as well as science in the practice of their profession. The want of the former, we can assure them, is of much more consequence than they imagine—often, indeed, of more real service, in so conjectural an art as ours, than attainments of much higher pretensions.

IV. The fourth chapter of Dr. Palmer's work is on the Influence of Moral Agents—of the Passions, &c. of the Mind, as the Exciting Causes of Disease.

In a work addressed to popular readers, the foregoing subject is capable, in proper hands, of being made highly interesting and useful. But Dr. Palmer's mode of handling it has disappointed us. An anatomical and physiological dissertation on the brain and spinal marrow is useless to the general reader, as being far above his comprehension, and not at all to his taste—while it is too superficial to be interesting to the physician or surgeon. In this, and in many other parts of the work, Dr. Palmer has shewn much of that want of tact and discretion which we have been just deploring. There are several sentiments confidently broached in this section, which we do not consider as drawn from accurate observation of facts—and which, indeed, are not such as we expected from Dr. Palmer. Thus, after telling us that the brain not only sympathizes with all the other organs, but is exposed to sources of irritation from which they are "altogether, or at least immediately, exempt,"—for instance, all moral and external impressions, strengthened by the refinement of education, and multiplied by the artificial wants and restless aspirations of civilization, he goes on thus:—

"And, at night, *when the heart pulsates, with an almost imperceptible languor, against the side*; *when the lungs perform, with inaudible effort and diminished frequency*, the process of respiration; and the stomach, having passed forward its evening meal, lies in a state of comparative inactivity and repose; *the brain, incessantly occupied in the work of nervous supply, is still exposed to agitation by every transient gust of passion and of feeling*,—still powerfully acted upon and excited by the mysterious imagery of dreams." 52.

Now our own observations have never shewn us this "almost imperceptible languor" of the heart in sleep. It may be sometimes a little more slow (and not often even that) in its action—but it is almost always more full and free—so that more rather than less blood is poured along the arteries at this time. In respect to the breathing, it is notoriously more deep and full, though somewhat less frequent, in sleep. As to its *inaudibility*, we believe that the exceptions to this are tolerably frequent—even in Dr.

Palmer's native land—and in that "house of God"—the Kirk. If the brain be "incessantly occupied in the work of nervous supply," Dr. P. ought to recollect that almost all the muscles of voluntary motion are at rest—and that there is an abdominal brain, and a ganglionic system, which afford no small aid to those involuntary motions that go on during sleep. How far the brain is agitated by "every transient gust of passion and of feeling," while we are asleep, we do not pretend to say.

The following passage is well written, though not perhaps quite so original as Dr. Palmer may think it to be.

"It has long been a favourite theme of argument and declamation with the moralist and philosopher, that the immoderate indulgence of sensual appetites and passions is invariably productive of punishment and remorse. *Yet no popular writer has hitherto arisen to point out the inference, which the preceding views evidently sanction, that disease and suffering are also the tax that man is doomed to pay for extraordinary elevation of intellect or acquirement.* Let them who seek to controvert this appalling truth, pause for awhile and survey the difference which exists between the man of talent, and feeling, and education, whose soul is exquisitely alive to every passing event,—to every impression of beauty and grandeur,—of deformity and imperfection, in the natural and moral world,—and the coarse and stupid being whose powers of locomotion alone distinguish him from the plant on which he gluts his craving appetite;—whose faculty of speech, from the inferior animal, which, in the fewness of his wants as in the simplicity of his attainments and diseases, he so closely resembles. Little elevated in the scale of intellect above the Cretin of the Alps, or the unsheltered savage of the southern ocean, the latter exhibits a physical condition happily distinguished by its hardihood and insensibility. He cannot feel those fearful paroxysms of mental agony and depression; he is insusceptible of that poignant and unutterable suffering,—which impart to the diseases of the sensitive and refined an additional bitterness and gloom; and, while aggravating the miseries of sickness, retard or utterly defeat the operations of medicine; and for which no splendour of acquirement or reputation, nor all the baubles of opulence, can afford to the wounded spirit a remedy or compensation. The stomach of the Boor may, indeed, be deranged by an occasional debauch. Extraordinary exposure to inclemencies of weather may induce in his hardy frame, an attack of rheumatism or inflammation. An emetic or a blood-letting will, however, usually suffice to restore his wonted health. To him are well-nigh unknown those more severe and embarrassing forms of nervous and intestinal disease which infest the higher orders of society. And, were he able to comprehend suffering which he has never felt, and thus to correctly appreciate his own superiority in physical enjoyment, he would, perhaps, regard the restless votary of fashion,—the busy aspirant to celebrity or wealth, with an eye rather of commiseration than of envy." 55.

Dr. Palmer is well aware that this subject has been touched upon by many writers. The following extracts are not unknown to Dr. Palmer.

"When the human species began to congregate in cities, it was soon perceived that in *this* class of society, the exertion of the *intellect* must predominate over that of the *body*. As civilization advanced, intellectual labour became more necessary, and the labourers multiplied in proportion. At the present period, the employment of a very large class of human beings, especially in civic life, consists almost exclusively in *mental* exertion. Look at the rulers of countries; the legislators, with their innumerable hosts of agents and sub-agents; the members of the pulpit; the bar; the medical world; the literary world; the superior orders of the mercantile world. In all these mental labour is the *regular duty,* and corporeal exertion only the *occasional relaxation.* Nay, in the vast body of mechanics and artists themselves, *thought* predominates over *action*. Even the semi-feminine man-milliner, who measures out our ribbon or lace, depends more on his talents, that is, on the volubility of his tongue, than on the agility of his muscles, for success in business. To such an extent is intellectual labour now arrived, that a very large and important class of society live entirely by 'teaching the young ideas how to shoot;' and a still larger class, who have no actual occupation, rack their minds with inventions, schemes, and projects that fade away as fast as they are engendered.

"Now I have before shewn that the more a voluntary *muscle* is exercised, within a reasonable limit, the stronger and more capable of exertion it becomes: it is so with the brain and nervous system; the more their faculties are brought into play, within a certain bound of moderation, the more extensive becomes the sphere of their power. The sense of touch, the sense of smell, and the sense of hearing, all become more acute, in proportion as they are exercised. *But this extra development and sensibility of the brain and nervous system, cannot take place but at the expense of some function, or structure, in the animal or organic system; for Nature, though sufficiently liberal, is, upon the whole, very economical of her gifts, and extremely impartial in the distribution of her favours. When, therefore, an undue share of the vital energy of any individual is directed to a particular organ or system, a proportionate subduction is made from some other organ or system, and this is a most undoubted, and a most important truth, which is little understood, and less attended to by the world in general.*"

"Civic life, by rendering the *senses* more acute, makes the *passions* more ungovernable than in rural retirement. In congregated masses of society, every kind of food for the passions is not only superabundant in quantity, but of the most stimulating quality. Hence, among a very considerable class in the upper walks of life, we find an unnatural and insalutary degree of excitement, kept up in the brain and nervous system from this prolific source. The extent of injury, which our health sustains in this way is beyond all calculation! Plato believed, that 'omnia *corporis* mala ab *anima* procedere;' 'all diseases of the body proceeded from the mind, or soul,' and certainly a great proportion of them do! Here we cannot fail to perceive the great analogy which obtains between the state of the digestive organs and that of the nervous system, in civic and luxurious life. The one is over-excited by too much and too stimulating *food*; the other, by excess in the *passions*. The derangements resulting from *each* set of causes act and react, directly or indirectly, on *both* systems; and thus it is that we never see a morbid condition of the *nervous system* unconnected with a similar condition of the *digestive organs*, and vice versâ.

"The *over-action* of the principal passions on the brain and nerves, closely resembles the over-action of food and drink on the stomach and other digestive organs, in many minute particulars, and especially by attracting an *undue proportion of blood* to the over-excited parts. The whole of the phenomena attending the Proteian host of *nervous diseases*, and all the most successful methods of treatment, attest that their immediate seat, or source, is an unequal distribution of the blood, and of the sensibility. The brain and nerves, becoming more irritable, from over-excitement by the passions, their vessels swell with blood, and this *local turgidity* causes a constant pressure on, and keeps up a perpetual irritation in, the whole nervous system."

"It is extremely difficult to draw a parallel of enjoyment and suffering, in the intellectual system between the upper and lower ranks of life. If, to undergo much pain for the sake of a little pleasure, be a proof that the balance is in favour of the *latter*, then the *beau monde* has it. But if, on the other hand, the Hindoo precept, that 'rest is preferable to action, sleep to waking, and death to all,' have any foundation in reason, then a question may arise, whether the lower classes of society, who have little susceptibility towards intellectual pleasures or pains, may not, upon the whole, claim the *balance* of enjoyment, in their journey through the present state of existence. But, at all events, Nature has here, as in most other instances, charitably ordained a surprising equilibrium. She has strewed the paths of rank, riches, and luxury, with a corresponding proportion of painful diseases, particularly of the nervous or intellectual system; while the uncultivated boor glides along, unconscious of the pleasures and unacquainted with the sufferings which necessarily grow out of civic society and intellectual refinement."*

We are afraid indeed that the aspiration in the last few lines of Dr. Palmer's extract will not soon be realized—namely, that the rustic boor will be brought to philosophize so wisely on the miseries of wealth and knowledge, and "his own superiority in physical enjoyment." The spreading thirst for information, and the rapid augmentation of numbers among the class of school-masters, militate sadly against Dr. Palmer's anticipations.

Dr. Palmer remarks that there is another peculiarity in the relative exercise of the different organs which, "has hitherto been suffered to pass without notice at all adequate to its practical importance," &c.

"*It appears to be a law of nature,—an invariable rule in the animal economy,—that no organ can be cultivated beyond a certain point, or long and powerfully exercised, except at the proportionate expense of its fellow members: that, in fact, if more than the due portion of nervous energy destined for its supply, be expended upon one, the other organs must experience a deficiency which will sooner or later be announced by the corresponding torpor or derangement of their respective functions.*" 56.

We have marked this passage in Italics, and if the reader will revert back to the passage marked in Italics, while quoting from a work published ten years previously, he will perceive he has copied it almost verbatim, and without any acknowledgment. He must indeed be conscious that this is not the only passage or sentiment he has made free with from the same source. But of this we do not complain. The practice is sanctioned by such high authority, now-a-days, as to excite no attention.

The melancholy effects of intellectual labour and corporeal inactivity in the poet, painter, sculptor, musician, man of letters, and statesman, are worked up by Dr. Palmer into a picture which, if generally perused, will drive half the above classes into the fens of Lincolnshire, (as we turn out horses into the marshes after the toils of the modern Babylon,) in order to take off all excitation from the intellectual system.

"Under these inauspicious circumstances, the intestinal canal, deprived of its wonted supply of nervous power,—of that invisible agent whose operation is as conspicuous as its nature unknown,—grows languid and irregular in the performance of its important functions. The muscular apparatus, if the excess be carried far, shares, ere long, the general failure. The body becomes feeble, emaciated, and unable to repel with pristine success, the encroachments of disease. The physiognomy assumes the peculiar characters of suffering and depression. And the brain itself, if original predisposition, or intemperance, favour not the development of the secondary affection, forms ultimately the seat of incurable alteration of structure. Hence the dreary assemblage of diseases, the indigestion, the intestinal tumour, and stricture, and ulceration,—the habitual congestions of the brain, the mental depression, the apoplexy, the madness

---

* Influence of Civic Life, &c, on Human Health and Human Happiness, 8vo, 1818.

and suicide,—of many of the most distinguished legislators and statesmen who have lately appeared on the theatre of the world. In illustration of this argument, it were almost superfluous to re-trace the sufferings and fate of the exiled Napoleon, the paralysis of the estimable Lord Liverpool;—the premature decease of the illustrious Pitt, of the lamented Canning;—the miserable doom of Romilly and of Castlereagh. If this melancholy retrospect suffice not for his conviction, let the reader turn and survey the character and fate of those master-spirits of the human kind, who have so signally adorned the literature of their age and country,—men, whose fervid genius, like that of the immortal Byron, while pouring splendour around their name, left the mind involved in wretchedness or gloom; whose intellectual light, while illustrating every object, and charming every eye around, preyed upon, and consumed, within, the powerful brain from which it emanated." 58.

Dr. Palmer has wisely referred also to those who expend their lives and fortunes in the gratification of their sensual appetites—the powers of whose systems are exclusively occupied in converting or throwing off the enormous quantites of highly-seasoned aliment which are requisite to stimulate a pampered appetite.

"The distended stomach loses, after a time, its wonted contractility and its energies. The brain becomes, at length, loaded from mere repletion; and its vigour and sensibilities gradually blunted and impaired. Acidity and indigestion, gout and jaundice, directly resulting from abuse of the stomach, and aggravated by the cerebral torpor consequent upon it, signalize the commencement of the ravages of disease: and effusion into the cavity of the chest, the bowels, or the brain, terminates an existence in which the moral qualities, however originally bright, have long been declining; and which the nobler passions and propensities, if ever they were developed, have well-nigh ceased to influence and adorn. Where the powers of the intellect habitually slumber, there will the stomach, in general, exhibit its greatest activity: and in proportion as the abuse of the digestive organs has been excessive, the torpor of the brain will be more strongly marked. Idiots are notoriously voracious · the stupidity of the city-alderman has long been proverbial." 59.

Dr. P. has not failed to notice the remarkable effects of strong *corporeal exercise* in obviating the evil consequences of inordinate indulgence and habitual plethora—acting, in short, like the safety-valve of the steam-boiler, in preventing the dangers of an explosion. Hence it is, that the eager sportsman, or the active man of business, may gorge and drink to an extent which would soon destroy the sedentary and literary classes of society. This immunity, however, is seldom perpetual—the hour of retribution, though tardy, comes at last. The great practical inference is, that we should (more easily recommended than practised) equalise the cultivation of the different organs and faculties—proportion the exercise of the mind to that of the body—avoid all inordinate anxiety and application of the intellect—all sedentary pursuits—sensual propensities—and extremes of every kind. No advice can possibly be better—provided it *can* be followed—and provided the individual *will* follow it! Some little knowledge of the world has taught us that not one in five hundred *can* adopt the judicious counsel of our author—and not one in five thousand *would* do so if they could. The public do not care one farthing about our harrangues against intemperance, indulgence, &c. They know, almost as well as we do, the evil consequences thereof. They do not go to the doctor for moral prohibitions against the *pleasurable* causes of diseases—No!—they go to him only for the cure of them when once induced. They can have sermons enough from the pulpit, and at a very cheap rate; but they regard admonitions very little till they come to suffer the penalties of transgression.

We were rather surprised to see introduced among the morbid affections "signally kept up and aggravated, by the operation of moral causes," IMPEDED ELOCUTION—or, in more vulgar phrase, STAMMERING. This Dr. Palmer divides into two distinct kinds, organic and functional. The former, of course, belongs to operative surgery—the latter to the physician—and, of late, to a distinct class of practitioners. As Dr. Palmer promises, in the course of a year, "a Philosophic Inquiry into the Causes, the Phenomena, and Treatment of Impeded Utterance," we shall not dwell on what he advances on the subject in his present work. If we may be allowed to form an anticipation from one of the following passages, we should advise the author not to publish his intended treatise—for assuredly, not one in ten thousand stammerers will ever be able to put in execution the only effectual remedy, according to Dr. Palmer's views. The first extract is very curious, and we know it to contain the truth.

"It is a very curious, and according to the writer's experience, an invariable fact, that, in defective articulation from a merely functional cause, the most inveterate stammerer, when alone or believing himself to be alone, can articulate without the slightest embarrassment or unnatural effort, and without particular attention to the process of verbal delivery. He can even speak, or read aloud, with the most perfect facility before a congregation of persons, however numerous: provided they are speaking at the same time; and he consequently feels that the attention of the assembly is not directed upon himself. But the moment the solitude of the stammerer is, in the one instance, broken in upon; or, in the other, the company, among whom he is declaiming, becomes silent, the brain loses its salutary control over the organs of voice and speech; and his progress is arrested. To persons unacquainted with the real nature of impeded articulation, this fact must, and does, appear perfectly inexplicable. Astonishment is frequently expressed at the facility and distinctness of utterance with which a notorious stammerer will accompany a congregation in the public performance of the services of his religion." 67.

The next extract which we shall introduce contains a melancholy prospect—the more melancholy, as we know that the talented author has long laboured under the defect which he so fully and eloquently deplores—and consequently is no bad judge of the difficulties which attend its eradication.

"*Medicinal Remedies* are not essential, as some interested writers have lately asserted, to the successful treatment of impeded utterance. Yet, skilfully selected and employed, they will accelerate the efficacy of a system of cure which rests upon comprehensive and philosophical principles. Thus, the embarrassment of articulation will be greatly relieved, and its removal assisted, by the prescription of tonic medicine, invigorating exercise, the shower-bath, and generous diet,—in fact, of every agent that is calculated to sustain or elevate the physical powers, and rouse the spirit of the stammerer from the state of morbid susceptibility and depression into which it is almost invariably plunged.

"Yet no physical treatment, however judicious and effective, will, of itself, permanently avail. Deep and bitter will be the disappointment of those who shall rely on it as a protection from the recurrence of their infirmity. A rigorous system of *Moral Discipline*,—long and unwearied exercise in concentration of the mind upon the process of speech, and in the practice of self-control, will be requisite to burst asunder the mystic links of morbid association, and effect a revolution in that moral state with which the evil habit is so closely interwoven. *Perfect freedom and fearlessness of mind, insensibility to the ridicule and the scorn of the ignorant and vulgar, a generous contempt for popular opinion, such an elevation of character and feeling,—such moral courage,—as a sense of moral purity can alone inspire,—constitute the goal to which the aspirations of the stammerer should be unceasingly directed.* This gained, recovery is no longer desperate. Every remaining obstacle will vanish before the auxiliary power of physical treatment. To achieve, therefore, his lasting liberation from the dreadful bondage of impeded utterance, *the individual should possess a mind not only strong in philosophy, but further sustained and enlightened by the moral virtues.* The sufferer may, perhaps, be cheered on and stimulated to increased exertion by the assurance that, while he incalculably promotes his own happiness and utility by this noble conquest of the infirmities of his nature; it will be impossible for him to acquire the necessary dominion over his feelings, and bring into due subjection his impetuous passions, without diminishing, at the same time, his susceptibility to the influence of the causes of disease, moral and physical; and thus rendering himself a more robust, a wiser, and a better man." 72.

We have taken the liberty of marking a few passages in the above extract in Italics. They will probably induce some readers to think that the able and estimable author of the work before us, is a little visionary in his expectations—or rather in his *moral* prescriptions; we say *moral*, because we have great reason to believe that, in *physical* prescriptions, Dr. Palmer is both energetic and judicious. We would ask the worthy author, how many, among his acquaintance in the midland counties—or any counties, north or south—are likely to attain the "perfect freedom and fearlessness of mind, insensibility to ridicule, &c." together with the "strong philosophy" and the "moral virtues," which he deems essential to the cure of stammering? To adapt his *methodus medendi* to the great mass of sufferers, Dr. Palmer must lower the scale of requisites by very many degrees—otherwise he will be like a great man, who had no defect of elocution—BURKE—

Too fond of the right to pursue the expedient.

Dr. Palmer next touches upon the "baleful influence of early (he ought to have said *too early*) mental exertion upon the physical development of children." We agree with him, that there exists not a more grievous or prevalent error than the solicitude which parents, now a days, evince, to stimulate the young mind to efforts injurious to the corporeal fabric. It is not, however, entirely the fault of parents. The "march of intellect,"—the doctrine that "knowledge is power"—the rapid increase of population—the closure of the flood-gates of war—the tremendous competition in all professions and avocations, force parents, school-masters, nay, Universities themselves, to stimulate youth to super-human exertions, by bribes, punishments, and every kind of positive and negative provocatives. The times are out of joint on this particular subject, and all the pullies and machines which our worthy author can employ will be of no avail in reducing the dislocation.

Passing over the 5th chapter on Inordinate and Defective Muscular Exertion, which does not contain any thing particularly worthy of notice, we come to the 6th, which has an attractive title in these days—"Of Intestinal Irritants considered as the Causes of Disease." The greater part of this long chapter, however, is occupied with the consideration of poisons introduced by accident or design, into the human stomach—intestinal worms—and the effects of dentition. These subjects are not treated in a manner to arrest the attention of the professional reader. The same may indeed be said of many other chapters in the work before us. We are tempted, however, to introduce an extract from the 7th chapter, in which Dr. Palmer anathematizes the CIGAR in terms somewhat akin to those employed by a Royal author (King James Ist.) in his learned "*Counter-Blast to Tobacco*," which he winds up by characterizing the use of this favourite plant as "a custom loathsome to the eye, hateful to the nose, harmful to the brain, dangerous to the lungs, and, in the black stinking fumes thereof, nearest resembling the horrible Stygian smoke of the pit that is bottomless."

Let us hear Dr. Palmer's opinion.

"The young man who, unjustified by the plea of ill-health, or unsanctioned by the prescription of his physician, has acquired the habit of smoking pipe or cigar, may assuredly congratulate himself on having reached the second stage of his progress from temperance to dissipation,—from elasticity of spirit and vigour of frame to premature imbecility and decay. As the reckless poacher is gradually led on, from his work of midnight depredation in the woods, to more daring acts of violence and rapine ; so will the youthful Smoker be too often insensibly allured from a wanton indulgence in the cigar to the sins of intoxication, and the ultimate sacrifice of his health, his character, and prospects. Let Parents, then, as they appreciate the responsibility which devolves upon them, solemnly protest against, and resist, the first encroachment of this pernicious habit in their family. Let the Women of England, whose influence is commonly as beneficent as irresistible, exert their powers in decrying the noxious practice, and averting from those in whose reputation and welfare they are so deeply interested, the moral pestilence. If the leaders of fashion in the land are resolutely bent on destroying the little remnant of energy and character which they still possess, let them pursue their ignoble propensities, and achieve the work of moral ruin as they are wont to dissipate their fortunes, in private. Society will be disposed to contemplate with singular philosophy and forgiveness, any act of moral suicide which these "Spoilers of the human hive" may be tempted to commit. But let them not contaminate with noxious exhalations the public atmosphere ;* nor the minds of the thoughtless and inexperienced, who are too frequently actuated by the vulgar ambition of aping fashionable follies, with their yet more pestilent example." 145.

Without at all advocating the cigar, and without knowing what the taste of it is, we cannot, from our own observations, join in this terrible denunciation of what we consider to be a very harmless, though perhaps not a very seemly indulgence. We do not believe that the present almost universal custom of cigar-smoking, among our youngsters, has any tendency to

* "The execrable practice of masticating Tobacco has not been adverted to : since it is now almost exclusively confined to the lowest orders. Cigar-smoking must be a more ancient custom than has hitherto been supposed : or the Poet, who, in the 16th century, so aptly depicted the strange spectacles constantly exhibited in the streets and thoroughfares of England at the present period, must have been endowed with the miraculous power of second-sight :

"There, in the twilight, stalks
"An apparition dire, breathing out flame,
"And wrapt in cloud of most unholy vapour."

the vice of drinking. We much doubt whether the cigar is not often a substitute for the other indulgence. Of this we are certain, that, on the Continent, where the cigar is in every one's mouth, there is very little disposition to drinking—all classes preferring the smoke of tobacco to the stimulus of wine or spirits. In this cold and damp climate—and in the malarious districts of warmer climes, we are disposed to think that a cigar is innocent—if not beneficial during the absence of the sun, and when dews and noxious exhalations are returning to the earth from whence they arose in the day. Upon the whole, we think Dr. Palmer's anathema against the cigar much too severe.

Under the head of marsh miasma, and its consequence, ague, we must notice a passage, which, we think, will bear us out in charging Dr. Palmer with a tendency toward the establishment of a system—or, in plainer terms, a HOBBY, equally as exclusive as that of the Abernethy school. Every one knows that if we direct our attention to a certain stage, and to certain phenomena of an ague, as for example, the flushed face, the cephalea, the throbbing carotids, &c. in the hot stage, we may very easily conjure up the idea of topical inflammation, especially if we keep out of sight, or in the back ground, the other stages of the disease, and the apyrexia between the stages. But who is not aware of the injurious consequences which would almost inevitably result from practice founded on this view of the malady? Let us see whether or not Dr. Palmer's doctrines would lead us into this snare.

"In all the cases of ague, which it has hitherto been the lot of the writer to examine, decided symptoms of cerebral congestion have marked both the commencement and progress of the affection: and the success of a curative plan, modified by these views, has been so prompt and striking as to strongly favour, if not decisively prove, the correctness of the observation."

"This treatment will be best exemplified by the detail of a case. Last spring a young tradesman had suffered long from ague, contracted during his residence in Lincolnshire. The paroxysm had, for some time, recurred at uncertain periods. The symptoms observed on the writer's first visit, Sunday noon, were principally, head-ach, loaded tongue, debility, great mental depression, scanty and unnatural discharges.—Prescribed, *a brisk emetic immediately; a full dose of Calomel at night, followed by a morning purgative: Ammonia in Camphor-mixture, on recurrence of the fit.—Diet: Tea, gruel.*—Monday. *Calomel and antimony, in large dose, every six hours; Acetate of Ammonia in the intervals; hair removed; cooling lotion to the head.* Tuesday. Head-ach intense, with violent throbbing of the carotid arteries. *Fifteen leeches to the temples; medicines continued: purgative next morning.* Wednesday. Head much relieved. *Medicines continued: blister to the nape of the neck, if the pain return.* Thursday. Countenance improved; tongue clean; skin moist; evacuations free and natural; mouth slightly sore. *Calomel discontinued: a purgative.* Friday. *Sulphate of Quinia, two grains every six hours; more generous diet.* Recovery, from this time, rapid. With the exception of slight rigors on the evenings of the second and third day, the paroxysm never re-appeared during the treatment: and has not, to the writer's knowledge, since returned." 149.

We see in the above case that ptyalism was induced, and we know that mercury will often stop the paroxysms of ague, at least for a time—but with all due deference to Dr. Palmer's judgment (especially as he professes to have seen very little of ague) we argue, that a much milder treatment than vomiting, purging, bleeding, "large doses of calomel and antimony every six hours," shaving the head, blisters, sore mouth—*and, after all, the quinine*—would have conducted the Lincolnshire visiter to a safe conclusion of his ague. On this point we appeal to the experience of our readers in general. The passage altogether exemplifies the effects of a predominant theory. That Dr. Palmer should embrace the cerebral doctrine of fever so long and so tenaciously maintained by Dr. Clutterbuck, is quite natural. The following curious *facts*, however, respecting the spontaneous origin of fever, and its subsequent reproduction by contagion, are worth several pages of theory.

"Within the last twenty years, the author has collected some curious facts respecting the origin and propagation of Typhus. From these, a striking example of its spontaneous commencement and obstinately infectious nature may be adduced. A robust young man, occupied in "ditching," near a healthy and finely-situated village in Staffordshire, was attacked with fever and pain in the limbs. The anti-inflammatory treatment was adopted. About the fifth day, without evident cause, the tongue became suddenly hard and black; the teeth covered with sordes; and the body with petechiæ. Delirium and subsultus ensued. He died next day. The mother, who had nursed him, sickened on the Monday, and expired on Wednesday morning, with symptoms yet more strongly marked. The fetor of her body before death was dreadful; after it, intolerable. Scarcely was life extinct when decomposition commenced; and, in a few hours, the corpse exhibited a mass of putridity. The house was immediately cleared of its inmates and furniture; white-washed, and fumigated under the inspection of the writer, and locked up. But the disease had already shewn itself among the children of an adjacent cottage; and from them been communicated to several persons in the village. The fever first shewed itself in early spring: autumn arrived ere it had completely disappeared. During the winter which followed, every vestige of it seemed extinct. But no sooner, in the ensuing spring, were the two deserted tenements re-occupied than typhus, of a milder character, was developed among their inmates, and again extended to the village. A second time was the disinfecting process more rigorously employed: and the houses shut up for many months. The fever again broke out on their being re-tenanted. Nor was the village completely freed from the scourge of typhus, until the two cottages from which the infection had originally emanated, were, at the suggestion of the writer, demolished." 157.

Our limits are in view, and we must skim very lightly over the remainder of the work. Upwards of 30 pages are occupied with the subject of hooping-cough. Dr. Palmer coincides pretty nearly with Dr. Webster in his theory of the disease. He admits, indeed, that hooping-cough consists originally and essentially in local, and probably specific, inflammation of the aerial mucous membrane—but that it speedily becomes "complicated with cerebral congestion—and *then* assumes the *convulsive* character." This is as much as to say that the *cough* depends on bronchial inflammation—but the *hoop*, on cerebral congestion. He goes just half way to Dr. Webster's theory. His ideas precisely accord with those of M. Desruelles, who proposes to designate hooping-cough by the term "*broncho-cephalitis.*" Nevertheless, Dr. Palmer takes a more rational view of the nature of the disease, (in our opinion) than either of the two authors above-mentioned. The following extract will convey some notion of Dr. Palmer's doctrine.

"During the violent paroxysms of the cough, the blood is propelled in undue quantity and with increased impetus to the brain; and the irritated and loaded organ re-acts with augmented violence on the local malady. Numerous facts in the history of Chincough are illustrated by this view. Of these, the most striking are,—the absence of the convulsive character in the other inflammations of the air-passages not complicated with cerebral irritation :—the existence of the cerebral symptoms invariably observed in even the mildest form of chincough :—the frequency of nasal hæmorrhage ; and the marked relief of the bronchial affection resulting from it :—the notorious tendency of the brain to active disease in hooping-cough ; *from which some writers have been led erroneously to infer that the latter is simply a cerebral affection* ;—and, lastly, the maintenance of the convulsive character long after every trace of the original inflammation of the respiratory membrane has disappeared ;* and the final removal of the disease, at that period, by spinal irritants, powerful moral impressions, or other agents which can exert no direct influence upon the bronchial membrane, or on any other organs, except the spinal marrow and the brain." 183.

Dr. Palmer reviews, in succession, the various remedies which have been or are used in hooping-cough—and his remarks are generally judicious—often acute and ingenious. Till the appearance of Dr. Watt's work on Chincough, in 1812—founded on illness and death in the author's own family, there was little satisfactory on record respecting the pathology of the disease—and very feelingly did Dr. W. deplore the absence of all precise information in the works which he consulted. On dissection of his own two children, bronchial inflammation was discovered—and other victims of the disease exhibited similar phenomena. In attributing it exclusively to this source, Watt was followed by Marcus, in 1816—Alcock in 1820—Guersent in 1823—Pearson in 1824—Dewees in 1825—Fourcade-Prunet in 1826—and many British periodical Reviewers. On the other hand, Leroy, in 1803 (Médecine Maternelle) Boisseau, Webster; and Begin contended that chincough is essentially a cerebral affection. Dr. Palmer thinks that the question has been decisively settled by Desruelles, whose doctrine has already been alluded to, but which may be concisely enunciated in the following passage translated by Dr. Palmer himself.

"Chincough is, in fact, only Bronchitis complicated with cerebral irritation. The inflammation of the bronchia is always primitive ; the irritation of the brain, consecutive. While the bronchitis is simple, the cough exhibits nothing peculiar ; but when the diaphragm and respiratory muscles, and those of the glottis and larynx,—are drawn into spasmodic action under the influence of the cerebral irritation, the cough changes its character and becomes convulsive." 183.

There is probably no material objection to this view of the pathology of hooping-cough. It is nearly that which we have occasionally urged when noticing Dr. Webster's theory—and it is a view which does not interfere with the most appropriate practice in this complaint.

In the 9th chapter of the work before us, Dr. Palmer has, under the head

---

* "This clearly explains one of the sources from which error has arisen in investigations of the Morbid Anatomy of Chincough. A child is destroyed by an affection of the brain, connected with the disease in its latter stages ; and consequently after every visible trace of the bronchial inflammation has disappeared. On dissection, the bronchial membrane is found in a natural condition : and hence an apparently correct although erroneous inference may be drawn that a morbid state of this membrane constitutes no essential character of Hooping-Cough."

of "Influence of the Atmosphere," given, among many other articles, a very interesting sketch of neuralgia, or tic douloureux, a disease to which Dr. P. has paid particular attention. We greatly regret that our closing limits prevent us from doing justice to this and the remaining part of the volume. We shall endeavour, however, to make room for a few extracts.

"In the *Treatment* of Constitutional Neuralgia, the obvious indications are, to relieve pain;—to avert or remove all those circumstances by which the distressing symptoms may be sustained or aggravated;—and to effect a permanent alteration in that state of the vascular and nervous systems which constitutes the proximate cause of the affection. Hence, the treatment may be distinguished into the Palliative, the Auxiliary, and the Essential Plans.—The *Palliative Remedies* are principally local. They consist in the application of leeches, of external warmth in the shape of fomentations, and currents of steam directed on the part; or stimulating or anodyne embrocations or plasters. They exert a merely temporary influence, either by diminishing the sensibility of the morbid nerve, or protecting from the impression of cold or of atmospheric changes, the surface on which it is distributed. They may be prescribed, with advantage, to procure respite from suffering until time has been allowed for the operation of more effective measures. From the application of blisters to the seat of pain, increased irritation, rather than relief, usually results. The internal employment of Opiates, as a mere Palliative, should also be avoided. By interfering with the biliary secretion, and constipating the bowels, it deranges the functions of the intestinal canal; and thus keeps up, or augments, the cerebral congestion and disorder from which the morbid affection has directly emanated.

"Under the *Auxiliary Treatment*, are comprehended an attention to, and the removal of, all those exciting causes, and conditions of the system, whereby the duration of the disease may be prolonged, or its severity aggravated. Respite of the brain from the fatigues and cares of business and from the influence of depressing passions;—defence or abstraction of the body from the operation of a damp or chilly atmosphere by clothing, or by confinement to a regulated temperature, or a change of residence:—and correction, by emetics, purgatives, or dietetic restrictions, of that unnatural state of the intestinal functions which, resulting from cerebral irritation, invariably accompanies Neuralgia,—are the principal means by which recovery may be accelerated. General blood-letting, although rarely essential, will also sometimes operate as a valuable palliative or auxiliary. By unloading the brain, it, under certain circumstances, not only mitigates pain and tranquillizes the perturbation of the system; but increases its susceptibility to the operation of more powerful and permanent remedies. In this, as in all other cases connected with cerebral congestion, blood will obviously be drawn, with greater economy and more decided effect, from the temporal or jugular vessels than from those of the arm.

"The great principle in the *Essential Treatment* of Neuralgia, is to restore the equilibrium of circulation, by unloading the vessels of the cerebral mass, or removing their morbid condition; and by giving additional energy and impulse to the enfeebled action of the blood-vessels on the surface, and in the extremities of the body. This object, Depletives and Counter-irritants, as Blood-letting and Blisters;—the more powerful Alteratives, as Mercury, Belladonna, and Arsenic; and Tonics, as Cinchona, Iron, and the Shower-bath, or a judicious combination or succession of several of these agents, will most effectually accomplish." 250.

General blood-letting is seldom required in neuralgia—leeches and cupping being usually sufficient. An antimonial plaster to the back of the neck is very serviceable.

"Of the *Alterative Remedies* in Neuralgia, *Mercury* is, doubtless, the most powerful and unerring in operation. Such, indeed, under ordinary circumstances, is the effect of this valuable agent that it will frequently accomplish the permanent removal of the disease, unaided by preliminary or consecutive treatment. Experience has, however, proved that, in the form of submuriate combined with opium, it exhibits an influence far more prompt and beneficent than when introduced only from the exterior by inunction, or internally without such combination. Calomel and Opium, in this as in several other morbid affections, appear to exert a peculiar—an almost specific—action on the system; which neither, seperately administered, is capable of producing. Many instances are on record, wherein as soon as the mouth has decidedly evinced the constitutional influence of the mercury, the Neuralgic paroxysm has suddenly subsided never to return.* Tonics, although not absolutely requisite, may subsequently be administered to repair the loss of strength induced as well by the remedy as by the disease. In very old cases, and under particular circumstances, of Neuralgia, the mercurial treatment, however judiciously or resolutely tried, will prove unavailing.†—The *Belladonna,* recommended and employed with such confidence and apparent success, in Neuralgia, by Mr. Bailey, has not realized, in other hands, the expectations which the testimony of this enlightened practitioner was calculated to inspire. In some instances, its effect in protracting, or lulling the tortures of the paroxysm, has been prompt and conspicuously marked. But the writer has yet no experience of its perfect or lasting efficacy, in solitary employment. The sufferings of the patient have sometimes indeed been rather aggravated by the cerebral confusion and terror consequent on the use of Belladonna, than relieved by its sedative operation.—*Arsenic,* although by no means invariably successful, is probably more to be depended on, in the treatment of Neuralgia, than Belladonna. Many estimable writers have attested its efficacy when preceded or accompanied by other remedies. But no instance of its permanent success, without such previous treatment or combination, has, hitherto, been observed by the writer. After local blood-letting, blistering, and mercury, the Arsenical Solution may frequently be prescribed in Neuralgia, as in Chronic Rheumatism and Intermittent Fever, with great and lasting benefit. This, therefore, may be regarded as constituting the precise period at which a trial of the remedy is correctly indicated. Its effect will be rendered more prompt and decisive by combination with a vegetable tonic." 252.

On the class of tonics Dr. Palmer makes some useful remarks. He observes, that the operation of invigorating medicines will invariably be assisted by the previous unloading of the brain and bowels. The quinine and carbonate of iron have proved the best tonics in our author's hands—but the "perfect success of either of these agents, prescribed alone, the present writer has repeatedly heard of but never yet witnessed." Dr. Palmer is disposed to attach more importance to a division of the nerve, or rather the dissecting out of a portion of it, than is now granted to the operation. Some cases by Dr. Warren of America, published in another part of this Journal, will support Dr. Palmer's views. The operation is only admissible at two periods—*first,* when the pain is no longer tolerable and menaces the patient's

* "See the evidence supplied by cautious deduction from numerous facts, in 'Observations on Neuralgia.' *New Medical and Physical Journal,* Vol. VII.

† "Two caseses of Constitutional Neuralgia in which the writer depended on Mercury alone, were promptly and permanently cured by it. Of six other cases, wherein it was employed in combination with blood-letting, blistering, and other remedies, four terminated successfully;—one exhibited only temporary relief; and one yielded neither to this nor to any other treatment."

life—*secondly*, when the local symptoms, from morbid associations, or change of structure, continue after the constitutional derangement from which they originally emanated, has been rectified—and the consequence survives the cause.

"This argument may be strikingly illustrated by a reference to the peculiar and only circumstances under which the operation of *Tapping* can with propriety be recommended in Ascites, or Abdominal Dropsy, consequent on diseased liver. The two following cases, selected with this view, will require no comment. A middle-aged man, of intemperate habits, was attacked with ascites and general dropsy complicated with an enlarged liver. The usual remedies had been administered in vain. At length, the accumulation of fluid became so great as to cause by its mechanical pressure on the stomach and diaphragm, constant rejection of food, and alarming difficulty of respiration. *Unless relief had immediately been obtained, the poor fellow must have inevitably sunk.* In order, therefore, merely to gain time, tapping was proposed; and more than fifty pints of serum drawn away. The fluid re-accumulated with such rapidity that the operation was again twice performed to *avert impending danger ere effective constitutional remedies could be brought into action.* Brisk mercurial purgatives and inunction, tonics, and diuretics, meanwhile, were sedulously plied; and fortunately began to tell just as the man had abandoned himself to despair. In a few days, he was completely unloaded by the natural outlets; and, twenty years subsequently, he exhibited all the characters of vigorous old age.—In the spring of 1824, a young woman, labouring under abdominal dropsy, consulted the writer. Her countenance and history indicated the existence or effects of diseased liver. The Surgeon who had treated the case with great energy and judgment, declared an opinion that he had gone far to remove the cause; but that the consequences would obstinately resist, as they had already done, all constitutional treatment. The liver disease had, in fact, been well-nigh cured; but the load of accumulated fluid was so great that the oppressed absorbents could not be brought to operate upon it. *Here the consequence obviously survived the removal of the cause.* After a few unavailing efforts to stimulate the absorbents by Mercury and Diuretics, a large quantity of limpid yellow serum was drawn off by tapping; and the disease has never reappeared." 256.

Here we must close our notice of Dr. Palmer's work. The title page—a "*popular*" work on medicine predetermined us not to review it at all, in conformity with our general custom. An examination of the book, (on account of the author's talents and character) convinced us that it was not at all adapted for popular perusal—or at least for popular comprehension—and the extracts which we have given will prove our judgment to be correct. Dr. Palmer *is* our personal friend—and *was* our former coadjutor in many a heavy day of literary labour. The profession will judge whether we have hesitated, on this account, to criticise—and that, even severely, when criticism appeared necessary. This is the way in which we treat our friends—and this is the way in which we wish to be treated by them in return. For the flattery of friendship and the abuse of enmity, we care about as much as AMRU, the MAHOMETAN GENERAL, cared for a certain gas which occasionally issued from the abdomen of the camel on which he rode. If Dr. Palmer possesses the mind which we believe him to possess, he will value this notice of his work in a proper light—and he will prefer a liberal and independent criticism to a fulsome panegyric. We advise him to give up all idea of *popularity*—to revise his work carefully—to address himself to his own profession, of which he is an honourable and distinguised member—and finally, to seek "the bubble reputation," neither in the "cannon's mouth," nor in the ephemeral periodicals of the day, but in the sober, settled, and permanent opinion of his medical brethren, who are the only legitimate judges of professional merit.

